# Supplementary material for: Social behavioral impairments in SYNGAP1-related intellectual disability
Source: Front Pediatr. 2023 Nov 29;11:1188117. doi: 10.3389/fped.2023.1188117 (PMC10716346; doi:10.3389/fped.2023.1188117)
Supplement: Supplementary file 1 [file Datasheet1.pdf]

Supplementary Information (SI)

Social Behavioral Impairments in *SYNGAP1*-Related Intellectual Disability

Journal of Autism and Developmental Disorders

Hajera Naveed, Maria McCormack, J. Lloyd Holder, Jr.

Affiliations: Department of Pediatrics, Division of Neurology and Developmental Neuroscience, Baylor College of Medicine and Jan and Dan Duncan Neurological Research Institute, Texas Children's Hospital

holder@bcm.edu

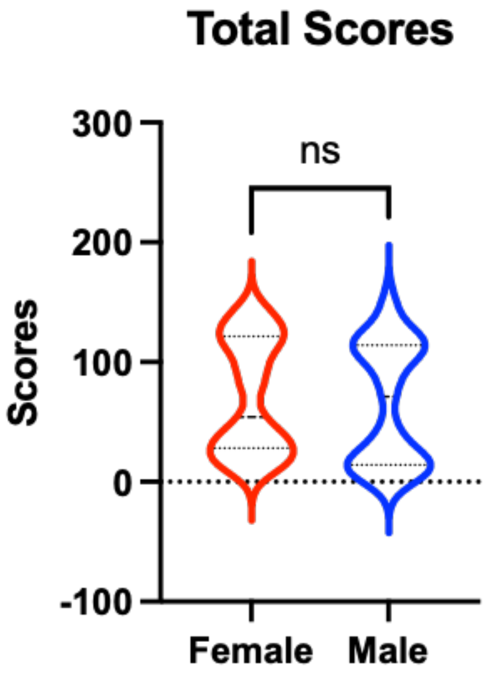

Figure S1 Total scores on full form for males (n=47) and females (n=45). Significance tested using a Mann-Whitney test (p = 0.2324)

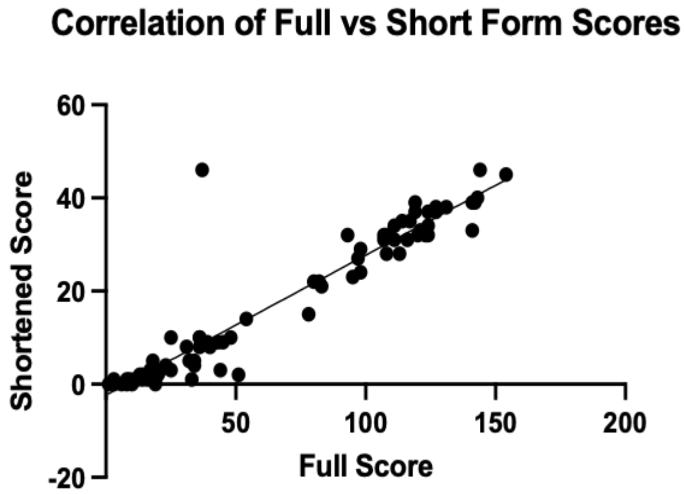

Figure S2 Simple linear regression of full form total scores vs short form total scores (n=81); r = 0.96
